# Cardiac MRI of differing ischemia and reperfusion times in a myocardial infarction pig model

**DOI:** 10.1038/s41598-025-11390-3

**Published:** 2025-07-19

**Authors:** David Boll, Simon Reiss, Heidi R. Cristina Schmitz, Christian Weber, Julien Thielmann, Felix Spreter, Kian Tadjalli Mehr, Diana Chiang, Markus Jäckel, Lukas Heger, Jonathan Rilinger, Dirk Westermann, Constantin von zur Mühlen, Timo Heidt, Michael Bock, Alexander Maier

**Affiliations:** 1 Department of Cardiology and Angiology, Faculty of Medicine, University Heart Center Freiburg-Bad Krozingen, University of Freiburg, Freiburg, Germany; 2 Division of Medical Physics, Department of Diagnostic and Interventional Radiology, Faculty of Medicine, University Medical Center Freiburg, University of Freiburg, Killianstraße 5a, 79106 Freiburg, Germany; 3 Center for Experimental Surgery, Faculty of Medicine, Medical Center University Freiburg, University of Freiburg, Freiburg, Germany; 4 Spemann Graduate School of Biology and Medicine (SGBM), Freiburg, Germany; 5 Faculty of Biology, University of Freiburg, Freiburg, Germany

**Keywords:** Myocardial infarction, Pig, MRI, CMR, Ischemia, Reperfusion, Preclinical research, Heart failure

## Abstract

The closed-chest porcine model of myocardial ischemia is an essential tool for preclinical research in cardiology. Current literature reports heterogeneous results regarding myocardial ischemia and reperfusion injury (IRI). This retrospective analysis presents our experience and cardiac magnetic resonance (CMR) findings while establishing a porcine IRI model. 8 male and 12 female juvenile German Landrace pigs received general anesthesia and antiarrhythmic pre-treatment. Myocardial ischemia was induced using transfemoral catheterization and balloon-occlusion of the left anterior descending (LAD) or the left circumflex (LCX) coronary artery under X-ray fluoroscopy guidance. The duration of ischemia varied from 30 to 90 min. CMR was performed at a clinical 3 T system including functional imaging, T1 and T2 mapping and late gadolinium enhancement (LGE) 2–5 h or three days post-reperfusion. Ventricular fibrillation (VF) was a recurrent event, which was counteracted with amiodarone, high blood potassium levels and in one case with propranolol. Males were more likely to experience VF than females and 15 of 20 animals survived the experiment. Resuscitation was required in 47% of the further analyzed animals. CMR revealed IRI in 14 of 15 animals in cardiac segments analogous to those supplied by the respective artery in humans. The mean left ventricular ejection fraction was reduced to 52% and 47% of animals had an impaired wall motion. Ischemia durations of 30–45 min did not result in LGE and in no significant T2 increase, whereas T1 was significantly increased (*p* = 0.02). Occlusion durations of 60–90 min resulted in LGE and T1 and T2 values both increased compared to 30–45 min of ischemia (*p* < 0.001 for T1 and *p* = 0.021 for T2). CMR of porcine myocardial IRI showed varying T1 and T2 patterns for different ischemia and reperfusion durations. For IRI, T1 elevation was the most sensitive marker. Significant myocardial infarction indicated by LGE only occurred after ischemic durations ≥ 60 min; thus, an appropriate occlusion duration must be chosen in pigs to create myocardial IRI for scientific studies.

**Supplementary Information:**

The online version contains supplementary material available at 10.1038/s41598-025-11390-3.

## Introduction

A reliable and applicable pig model is an essential tool for translational cardiovascular research^1^. Pigs closely represent human cardiac anatomy, function and response to ischemia^1–3^. Several research groups have established mainly two major pig models for myocardial infarction (MI), a closed- and an open-chest MI model. The choice of model should be guided by the specific scientific question. The closed-chest model, in which a coronary artery is occluded via balloon inflation via minimal invasive arterial access, closely mimics the pattern of tissue injury and clinical presentation of AMI in humans. The open-chest model with surgical coronary ligation requires thoracotomy and pericardial opening, which alters hemodynamic parameters and is more traumatic^4,5^. This model offers advantages such as direct access to the heart for procedures like intracardiac injections and myocardial biopsies. Comparable arrhythmia and mortality rates have been reported for both open- and closed-chest models, whereas findings on infarct size and cardiac function remain conflicting^4–7^. Other methodological modifications, such as the duration of ischemia, differing anesthesia protocols and mortality rate variations remain major challenges in both models^7–10^. This can lead to conflicting published results for MI pig models.

CMR has an established role in current ESC guidelines for acute coronary syndrome and cardiomyopathies^11,12^. It provides high-resolution information on cardiac structure, function and tissue composition^13–18^. T1 and T2 weighted sequences along with LGE are standard methods for the detection of myocardial edema and necrosis after MI^19–21^. They facilitate the clinical assessment of left ventricular remodeling, area-at-risk (AAR), and infarction size (IS), which serve as prognostic parameters after AMI^11,23,24^. Additionally, CMR holds potential for real-time guidance of cardiac catheterization procedures^25–27^. T1 and T2 mapping have emerged as quantitative methods for post-MI imaging and help to establish contrast agent-free CMR protocols^28,29^. They offer reproducible tissue characterization with an improved sensitivity for edema, intramyocardial hemorrhage and microvascular obstruction (MVO)^20,30–33^.

In this study, we share our experience in establishing the current standard porcine MI model at our facility and present CMR findings shortly after IRI. This was motivated by varying published results and protocols for myocardial infarction pig models. The primary aim was to characterize myocardial tissue and identify nonviable myocardium using CMR following varying durations of coronary occlusion. Additionally, we evaluated factors related to arrhythmia in this model. Finally, we discuss the results in the context of existing MI protocols and CMR findings in porcine studies.

## Methods

### Animals

Juvenile German Landrace pigs (Breeder: Franz und Thomas Lais GbR, Hartheim-Bremgarten, Germany) with a bodyweight (bw) of 50–70 kg were transported to the Center for Experimental Models and Transgenic Service (CEMT) in Freiburg approximately 10 days before the start of the experiments. At the CEMT, the pigs were fed a maintenance diet twice a day and had access to water ad libitum. The pigs underwent preoperative fasting for at least 12 h before the experiments.

### Experimental design

Myocardial IRI was induced in 20 pigs following the protocol outlined below. After successful induction, 10 animals underwent CMR 2–5 h post-reperfusion, while 5 animals underwent CMR three days after reperfusion. Thus, CMR of 15 animals was analyzed. The mean duration of coronary occlusion across these animals was 44 ± 15 min. 12 animals were subjected to an ischemia of 30–45 min (37 ± 4 min) duration, referred to as short ischemia group, and in three animals the ischemia lasted for 60–90 min (70 ± 17 min), referred to as long ischemia group.

### Myocardial ischemia and reperfusion pig model

**Fig. 1:**
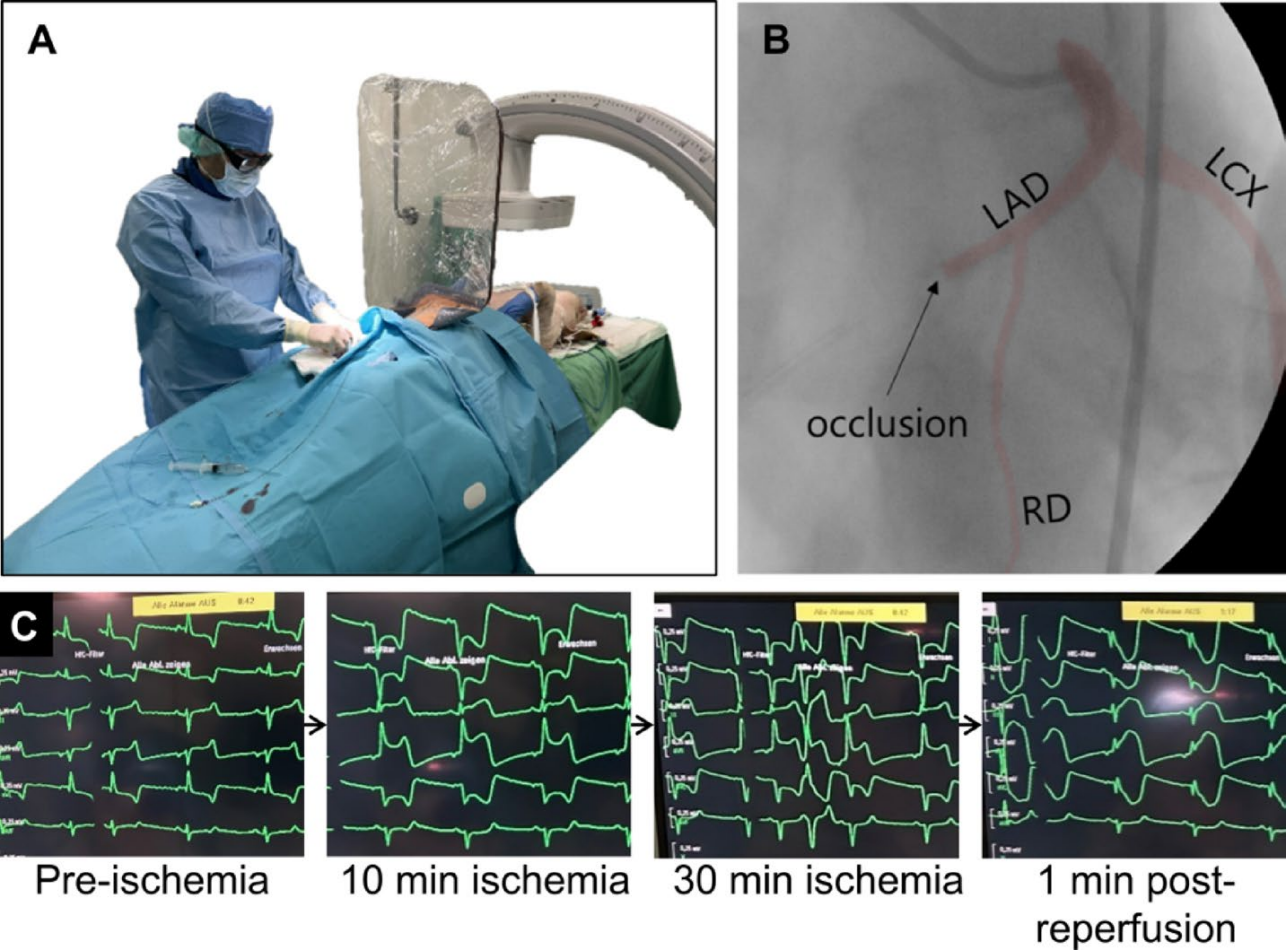
Induction of myocardial ischemia reperfusion injury. (**A**) Interventional setup with an angiography x-ray system for catheter-based balloon occlusion of a coronary artery. (**B**) Coronary angiogram showing the left coronary artery (highlighted in red) with the LAD occluded downstream of the origin of the first ramus diagonalis (RD). The estimated position of the balloon for an LCX occlusion is indicated by “*”. (**C**) Typical progressive ECG changes during LAD occlusion: In the initial pre-ischemic state, a sinus rhythm without signs of ischemia. Within the first 5–10 min of ischemia, ST-segment elevations > 0.1 mV and reciprocal depressions emerge, becoming progressively more pronounced over time. Ventricular ectopic beats frequently appear after approximately 30 min of ischemia. Immediately following reperfusion, pronounced T-wave changes are evident.

Anesthesia was induced via intramuscular injection of midazolam (0.5 mg/kg bw) and ketamine (20 mg/kg bw). Following establishment of intravenous (IV) access via a peripheral ear vein and orotracheal intubation, the animals were further anesthetized by IV administration of propofol (2–4 mg/kg bw) and transported to the X-ray fluoroscopy facility (Fig. 1A). Subsequently, anesthesia was switched to inhalation anesthesia using a Dräger Fabius Trio workstation (Drägerwerk AG & Co. KGaA, Lübeck, Germany). CPPV was applied with a mixture of isoflurane (1.5–2 Vol%) and oxygen/air (FiO2 0.3–0.4). Animal monitoring included continuous six-lead electrocardiography, pulse oximetry, blood pressure and respiratory parameters, which were regulated by adjustment of mechanical ventilation.

Inhalation anesthesia was accompanied by IV administration of vecuronium (0.2–0.4 mg/kg bw per h) and fentanyl (0.002–0.004 mg/kg bw per h) to ensure muscle relaxation and analgesia. Ringer’s solution (with added KCl, Mg and glucose) was administered at a rate of 8–10 ml/kg bw per h to regulate hydration status. To reduce susceptibility to ventricular fibrillation (VF), the pigs received a 300 mg IV dosage of amiodarone before catheterization. One animal (#15) was additionally pretreated with 2 mg propranolol. Heparin (100 IE/kg bw bolus IV, then 50 IE/kg bw every two hours into the femoral artery) was administered to prevent clotting and thromboembolic events during catheterization.

An 8F femoral artery access sheath was percutaneously introduced into the right common femoral artery using the Seldinger technique. Arterial blood gas analysis was then performed to measure the potassium concentration. Up to 10 ml potassium chloride (1 M) was administered IV stepwise to raise the commonly low potassium concentration in the blood to approximately 5 mmol/l, further reducing the risk of cardiac arrythmia. A coronary guiding catheter (Tiger 6 Fr, Terumo) was advanced through the aorta and engaged into the left coronary ostium. A flexible guide wire (0.014″) was then positioned in either the LAD or the LCX, and a balloon tipped catheter (3 × 12 mm) was advanced over the wire and positioned between the first and second diagonal branch for the LAD or the mid LCX. Iodine-containing contrast medium released from the catheter tip was used to visualize coronary blood flow and to identify coronary branches. Inflation of the balloon (6–11 bar) occluded the coronary artery and completely stopped coronary blood flow distal to the balloon, as confirmed by contrast medium infusion (Fig. 1B). Acute myocardial ischemia was further indicated by typical electrocardiographic alterations (Fig. 1C). After occlusion the balloon was deflated to allow for passive reperfusion, which was confirmed using contrast medium application.

In cases of VF, coronary occlusion was usually terminated. In pig #14, however, the occlusion was continued after rapid and successful resuscitation. Then, cardiac massage and electric cardioversion (200 J, biphasic) were applied until a sinus rhythm could be detected. Boli of amiodarone (300 mg) and adrenalin (1 mg every 3–5 min) were IV administered during resuscitation. After withdrawal of the catheters, wires and the sheath, inhalational anesthesia was discontinued and IV anesthesia with propofol was restarted.

**Fig. 2:**
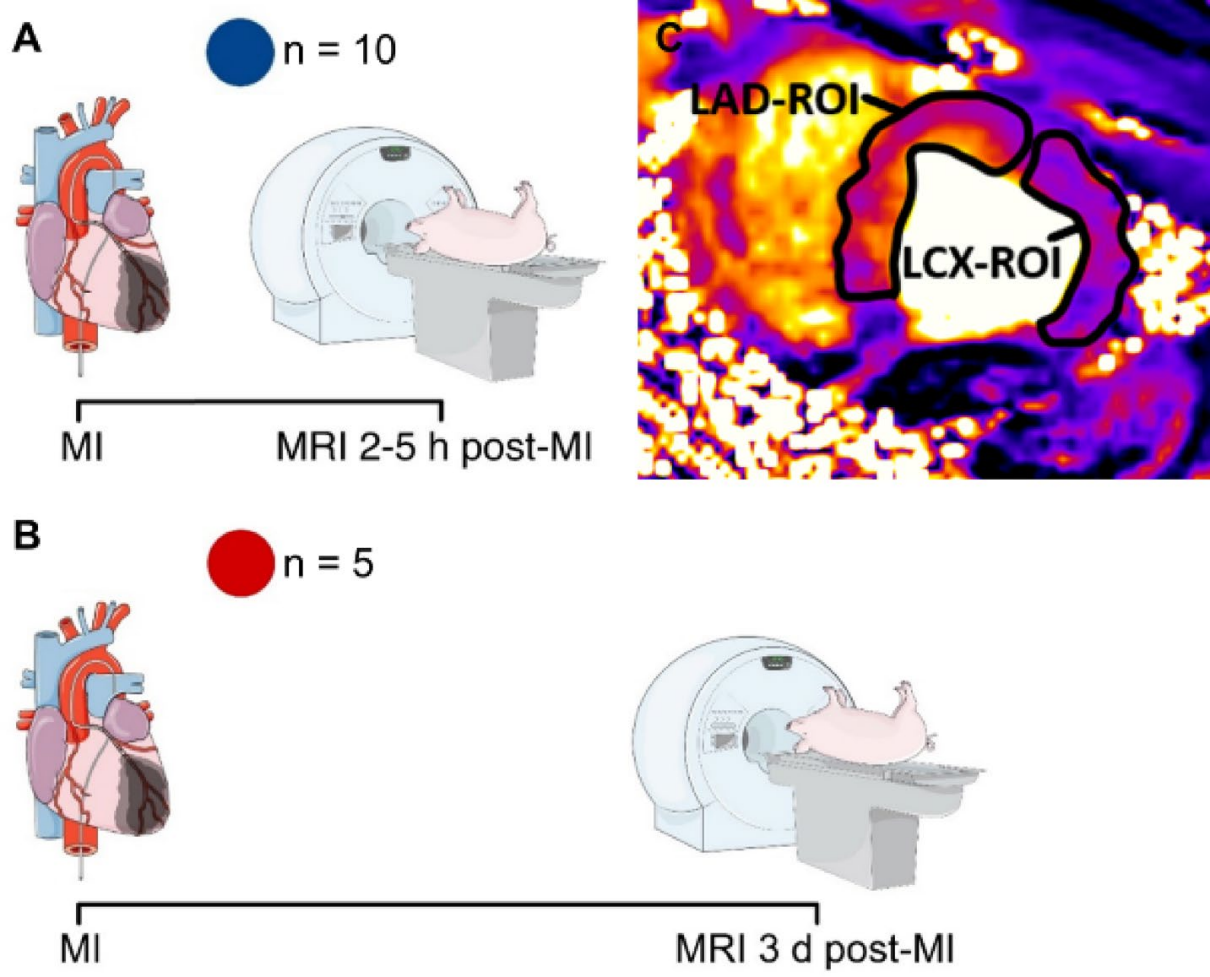
Experimental design. (**A**) Blue symbols represent pigs that underwent MRI 2–5 h post MI-induction. (**B**) Red symbols represent pigs that underwent MRI three days post MI-induction. (**C**) Representative placement of the LCX and LAD ROIs.

At this point animals were assigned to one of two groups. Animals of the first group were directly transported to the clinical MRI for CMR (Fig. 2A). Animals of the second group were transferred back to the CEMT animal facility, where the emergence from anesthesia was initiated. The conscious animal was further supervised by experienced veterinary staff. Three days after IRI, the animal was again anesthetized using the same protocol as described above and then transported to the clinical MRI for CMR (Fig. 2B). After MRI, all animals were euthanized in deep anesthesia by IV injection of potassium chloride (2 mmol/kg bw).

### Cardiac MRI

Cardiac MRI was performed at a clinical 3 Tesla MRI system (PrismaFit, Siemens). During MRI, anesthesia was maintained with IV propofol (8–15 ml/kg bw per hour) accompanied by IV vecuronium (0.2–0.4 mg/kg bw per hour) for muscle relaxation. Mechanical ventilation with oxygen/air (FiO2 0.3–0.4) was applied and heart rate and rhythm were monitored using the four lead ECG provided by the MRI system.

The animals were imaged in head-first supine position with the heart at the magnet iso-center. The 32-channel spine coil and a flexible 18-channel thorax coil were used. All images were acquired during manually induced breath-holds and gated to end-systole. After an initial set of localizer images, functional and volumetric data was acquired using a multi-slice 2D cine bSSFP sequence in short-axis view (TE/TR = 1.5/3.0 ms, flip angle (FA) = 42°, BW = 970 Hz/px, FoV = 340 × 273 mm^2^, matrix: 224 × 126, slice thickness (SL) = 8 mm, number of slices: 6–8, retrospective cardiac gating with 20 reconstructed phases, multiple breath-holds). After functional imaging, T1 and T2 maps were acquired in three or four mid to apical short-axis slices, depending on the size of the heart. For T1 mapping, an inversion recovery bSSFP sequence was used (TE/TR = 1.2/2.7 ms, FA = 35°, BW = 1085 Hz/px, FoV = 360 × 307 mm^2^, matrix: 192 × 132, SL = 5 mm, 8 different TI values from 100 to 4500 ms, depending on the heart rate, single breath-hold). T2 mapping data was acquired with a T2-prepared FLASH sequence (TE/TR = 1.3/3.1 ms, TE_T2prep_: [0, 30, 40] ms, FA = 12°, BW = 1185 Hz/px, FoV = 360 × 247 mm^2^, matrix: 192 × 132, SL = 6 mm, single breath-hold). The vendor-provided inline motion correction and calculation of T1 and T2 maps were used for image post-processing.

Late enhancement was performed 10 min after IV injection of 2.5 mmol/kg Gd. The optimal inversion time was determined based on a short-axis TI scout measurement. Sequence parameters were set to TE/TR = 1.4/3.7 ms, FA = 20°, BW = 465 Hz/px, FoV = 3602 mm^2^, matrix: 144 × 141, SL = 10 mm, single breath-hold.

### Image analysis

Wall motion abnormalities were assessed visually on cine images. A segment’s wall motion was deemed impaired if myocardial contraction was reduced. The left ventricular ejection fraction (EF) was measured using the syngo.Via software provided by the MRI system. Phase-sensitive inversion recovery (PSIR) and magnitude inversion recovery (MAG) LGE slices, as well as T1 and T2 maps, were visually evaluated for regions with enhanced values. Location and extension of changes were described. The visual three-dimensional extent of the LGE was analyzed using the left ventricular 17-segment model recommended by the American Heart Association (AHA)^34^. Segment 17 (apex) could not be assessed due to missing long-axis images.

Additionally, for each animal an apical T1 and T2 short-axis slice were quantitatively evaluated in two ROI: one containing LV myocardium primarily supplied by the LAD and the other by the LCX (Fig. 2C). Thus, one ROI represented myocardium with IRI, while the other represented healthy remote myocardium. In the ROI the mean and standard deviation of T1 and T2 were determined for each animal. The difference in the mean T1 between ischemic and remote myocardium was defined as T1 = T1 (ischemic) − T1 (remote), and the same procedure was applied to the T2 maps.

### Statistical analysis

Statistical analysis was conducted with Prism 10 (GraphPad Software, USA). Results were ruled significant for *p* < 0.05. Differences were analyzed between shorter and longer ischemic periods. In addition, the subgroup of animals that underwent CMR three days post IRI-induction was analyzed separately. Mean ΔT1 and ΔT2 values and standard deviations of groups were computed from the individual differences. Distributions of the samples were tested for normality using the Shapiro–Wilk test. If the samples were distributed normally, groups were compared using an unpaired, two-tailed t-test. If samples were not distributed normally or the sample size was < 3, groups were compared using a Mann–Whitney U test. The Pearson correlation coefficient r was calculated to detect correlations between the occlusion time and mapping values.

## Results

### Juvenile German Landrace pigs were prone to VF during myocardial ischemia

VF occurred in 13 of 20 animals with IRI, causing five deaths (75% survival).

**Fig. 3:**
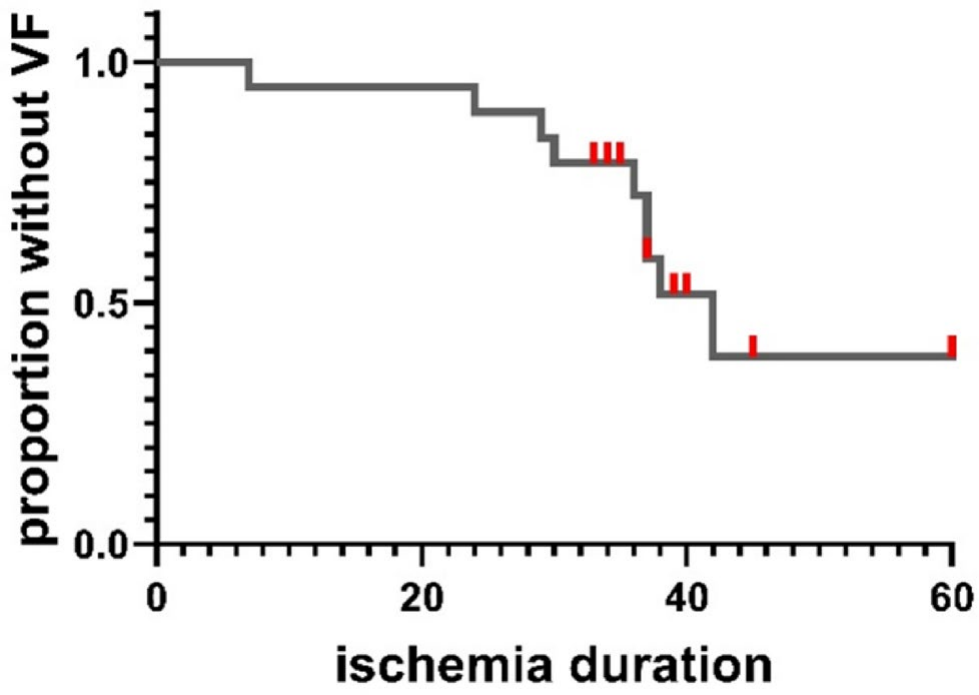
Time to VF during coronary occlusion. The Kaplan–Meier curve (19 pigs) illustrating the time to first occurrence of VF during coronary occlusion. Red censor marks indicate pigs where ischemia was terminated before VF occurred. VF before or after coronary occlusion isn’t displayed.

Most VF occurred during coronary occlusion, after a mean ischemia duration of 31 min (Fig. 3). VF occurred in all cases with ischemia ≥ 40 min, except for pig #15 (90 min LAD occlusion), which was pretreated with propranolol.

VF appeared immediately after reperfusion in two animals and post-CMR in pig #9. It also occurred before occlusion in one animal, resulting in a shorter subsequent ischemia of 10 min. This animal was excluded from CMR analysis. Among the 15 animals included in the CMR analysis, seven (47%) were resuscitated.

Males were more likely to develop VF than females (7/8 males vs. 6/12 females) and LAD occlusion triggered VF more frequently than LCX occlusion (5/7 vs. 7/12).

### LAD supplied septal and anterior LV wall, LCX supplied lateral and posterolateral LV wall in German Landrace pigs

**Fig. 4:**
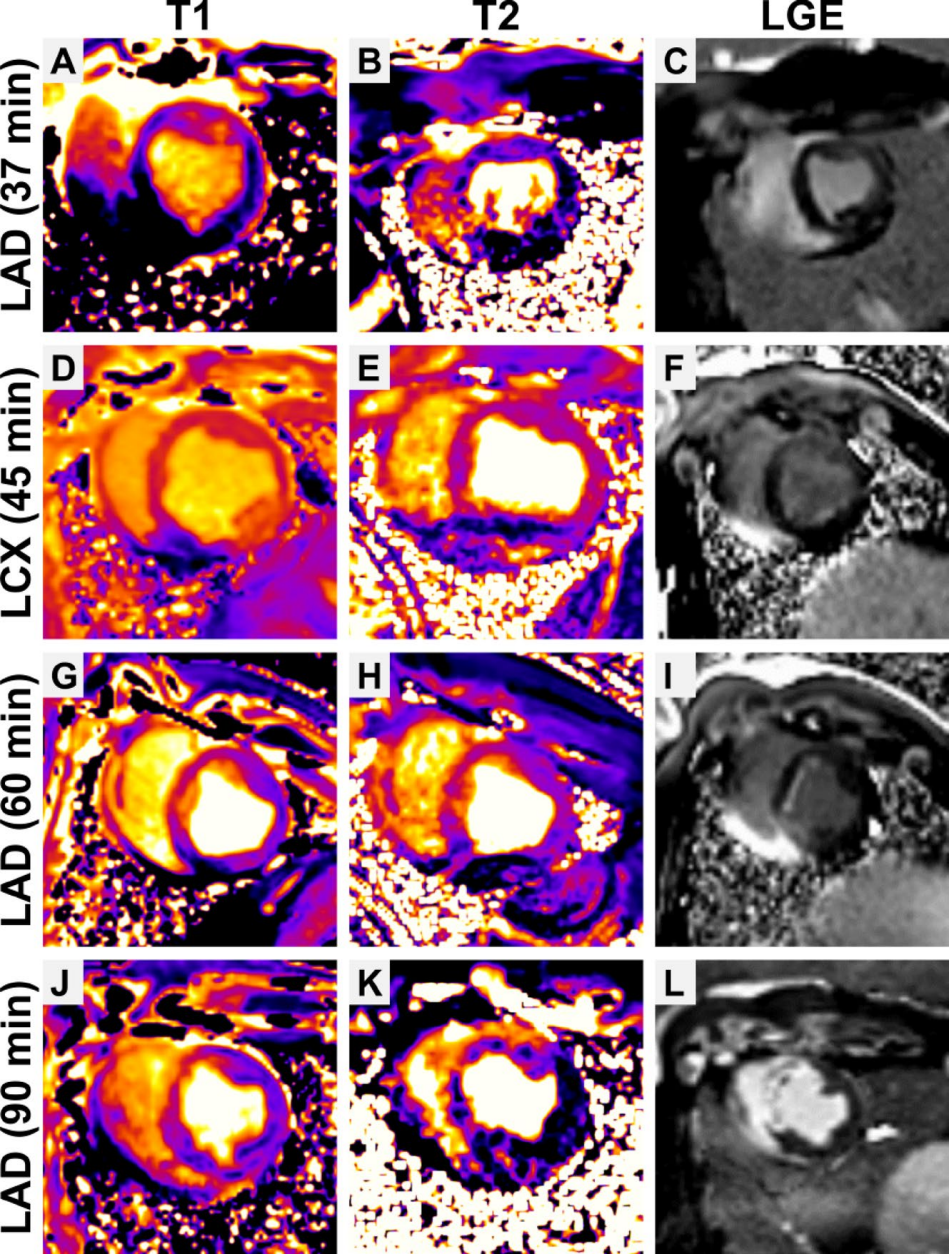
Representative T1 map (left), T2 map (middle) and LGE (right) images. (**A**–**C**) CMR images acquired 2–5 h after a 37 min LAD occlusion. (**D**–**F**) CMR images acquired three days after a 45 min LCX occlusion. (**G**–**I**) CMR images acquired three days after a 60 min LAD occlusion. (**J**–**L**) CMR images acquired three days after 90 min LAD occlusion.

CMR revealed visual signs of IRI in 14 of 15 animals (detailed data is provided in Supplementary Tables 1–4). LAD occlusion affected the anteroseptal, inferoseptal and anterior LV walls, whereas LCX occlusion impacted the lateral and posterolateral LV walls (Fig. 4).

### LVEF and wall motion after myocardial ischemia

**Fig. 5:**
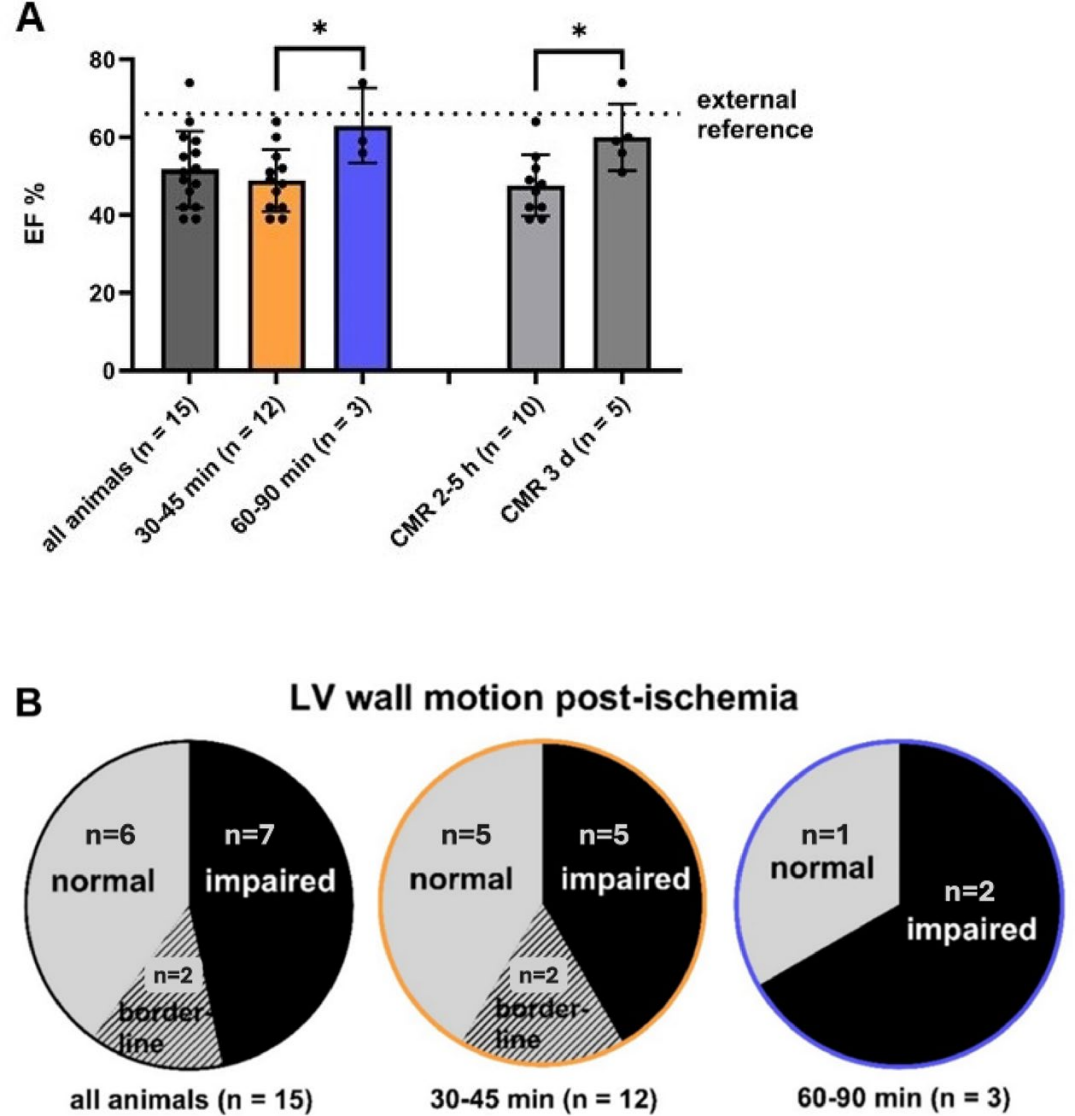
LVEF, wall motion alteration. (**A**): Overall mean EF measured post-ischemia was 51.7 ± 10%. The EF following short ischemia was significantly lower compared to long ischemia. The EF measured 2–5 h post-ischemia was significantly lower compared to three days post-ischemia. The dashed line indicates the EF in healthy pigs of similar age and breed^55^. (**B**) Wall motion alterations observed post-ischemia.

The mean EF across all 15 pigs was 52 ± 10%. Pigs with long ischemia had a significantly higher EF than those with short ischemia (*p* = 0.02). EF immediately post-ischemia was lower than at three days post-ischemia (*p* = 0.015, Fig. 5A).

Wall motion was impaired in seven, borderline in two, and normal in six animals. Some showed hyperdynamic LV function. Impairment was more frequent in long ischemia than short ischemia (Fig. 5B**)**. This was also seen in the group assessed three days post-ischemia (Supplementary Table 2).

### LGE was observed following 60–90 min of ischemia

Of the 12 animals with ischemia durations < 45 min, none displayed clear LGE (Fig. 4C & F). In contrast, 60 min of ischemia caused subendocardial infarction in LGE imaging, subtle in pig #13 and pronounced in pig #14 (Fig. 4I). 90 min of occlusion led to a transmural infarction in pig #15 (Fig. 4L).

LGE consistently affected segments 8, 13 and 14 of the AHA 17-Segment model. Additionally, segment 2 was involved in animal #13.

### T1 mapping changes were observed after short and long ischemia durations

In 86% of the animals regional brightening was visually observed in T1 (Supplementary Tables 1 & 2).

**Fig. 6:**
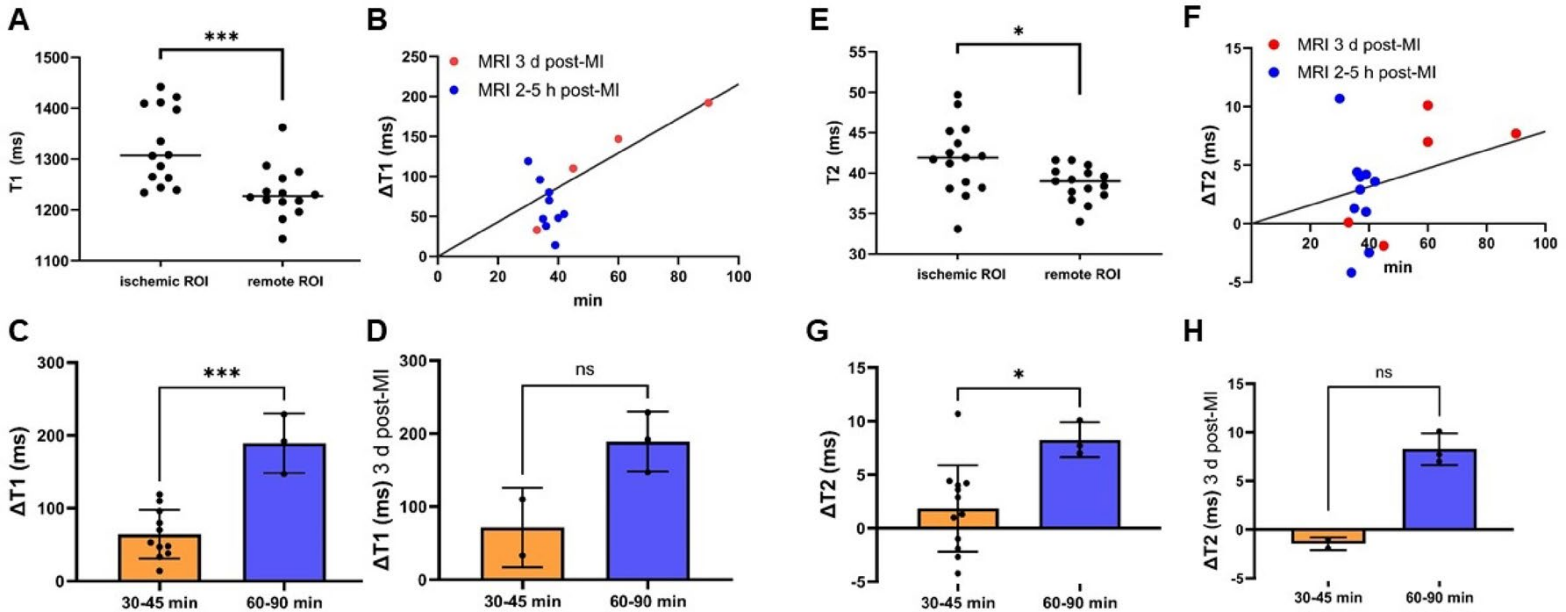
T1 & T2 mapping results. (**A**) T1 values of ischemic myocardium compared to remote myocardium. (**B**) T1 for different infarct durations with linear regression. (**C**) T1 grouped for different infarct durations considering all animals. (**D** T1 grouped for different infarct durations considering only the five animals with MRI three days post-MI. (**E**) T2 values of ischemic myocardium compared to remote myocardium. (**F**) T2 for different infarct durations with linear regression. (**G**) T2 grouped for different infarct durations considering all animals. (**H**) T2 for different infarct durations considering only the five animals with MRI three days post-MI. ‘Ns’ denotes *p* ≥ 0.05; *p < 0.05, **p < 0.01, ***p < 0.001.

Quantitative analysis (Table 1) showed significantly greater T1 times in ischemic compared to remote myocardium in apical locations (*p* = 0.001), regardless of ischemia duration (Fig. 6A). There was a strong positive correlation between ischemia duration and T1 (Fig. 6B), with significantly greater ΔT1 after long ischemia (*p* = 0.0001, Fig. 6C).

**Table 1:** Mapping analysis after myocardial ischemia.

Group	Parameter	T1		T2	
All animals	Remote	1235 ± 52 ms		38.7 ± 2.1 ms	
	Ischemic	1326 ± 76 ms		41.8 ± 4.4 ms	
	ΔT = ischemic − remote	91 ± 63 ms	*p* = 0.001	3.1 ± 4.5 ms	*p* = 0.019
	Correlation: ischemia duration & ΔT	r = 0.73		r = 0.43	
	ΔT (short ischemia)	64 ± 34 ms	*p* = 0.024	1.9 ± 4.0 ms	*p* = 0.19
	ΔT (long ischemia)	189 ± 41 ms	*p* = 0.001	8.3 ± 1.6 ms	*p* = 0.026
	ΔT (long ischemia ) − ΔT (short ischemia)	125 ms	*p* = 0.0001	6.4 ms	*p* = 0.021
Animals with CMR three days post ischemia	ΔT	142 ± 76 ms	*p* = 0.001	4.4 ± 5.5 ms	*p* = 0.73
	Correlation: ischemia duration & ΔT	r = 0.76		r = 0.73	
	ΔT (long ischemia)ΔT (short ischemia)	125 ms	*p* = 0.2	9.7 ms	*p* = 0.2

ΔT = difference in mean T1/2 between ischemic and remote myocardium, long ischemia = animals with 60–90 min of ischemia, short ischemia = animals with 30–45 min of ischemia.

This trend persisted in the subgroup that underwent CMR three days post IRI-induction: ischemic myocardium exhibited higher T1 values, and ΔT1 increased with ischemia duration (Fig. 6D).

### T2 changes paralleled LGE findings

Regional T2 increase was visible in 60% of animals (Supplementary Tables 1 & 2).

Quantitative analysis (Table 1) revealed greater T2 values in ischemic compared to remote myocardium in 80% of animals (Fig. 6E). In similarity to LGE, the T2 increase was only significant in the long ischemia group (*p* = 0.026). There was a moderate positive correlation between ischemia duration and T2 (Fig. 6F), with ΔT2 being significantly greater after long ischemia (*p* = 0.021, Fig. 6G).

In the subgroup examined three days post-MI, this trend was also observed: ischemic myocardium showed greater T2 values, and ΔT2 correlated positively with ischemia duration (Fig. 6H).

## Discussion

### Porcine MI model

In our study, both the LCX and LAD were targets of occlusion. Thereby systematic measurement errors caused by MRI artefacts, especially susceptibility-induced signal voids in the posterior myocardium, were minimized. The areas supplied by the occluded arteries mostly corresponded to the regional distribution in humans. This underscores the similarity in blood supply of the myocardium between pigs and humans^3,35^.

The survival rate of 75% is expected based on available literature^7,36^. However, reported survival rates vary widely, ranging from 59 to 97%^37,38^. Mortality was mainly associated with VF during ischemia and in case of VF, coronary occlusion mostly had to be terminated. Therefore, antiarrhythmic therapy is essential in this model^6^. The antiarrhythmic measures applied in our study, i.e. amiodarone, KCl^39^ and propranolol, provided adequate protection and differed from the strategies described in other protocols. Improved survival may be achieved through intracardial defibrillation catheters^2^.

On the one hand, VF occurred more frequently in LAD than LCX occlusions, which is supported by literature^6^. On the other hand, choosing the LAD over the LCX yields a more exactly defined balloon positioning, more available literature and a functionally and clinically more relevant MI^40,41^. Sex is recognized as a significant factor in experimental heart studies^42^. In our study, female pigs tended to be more resistant against the occurrence of VF. Almost all surviving males were resuscitated, which heavily impacts study targets. Published data suggest electrophysiological differences between male and female pigs, for example in post-ischemic atrial fibrillation^43^. However, IS and MVO were not influenced by sex of pigs in a recent study^44^.

Clinical guidelines recommend the early IV administration of beta-blockers, preferably metoprolol, in the context of AMI^11^, based on the cardioprotective and antiarrhythmic effects of beta-blockers^45–48^. A porcine study reported a 27% reduction in IS when metoprolol was administered prior to reperfusion^49^. This effect has been attributed to its unique changes in neutrophil dynamics^45,50^. Conversely, propranolol did not show the same reduction in IS, but has been associated with a reduced risk of arrhythmia and sudden cardiac death in AMI^51^. In this study, propranolol was administered to one pig (#15) with the intent to leverage its antiarrhythmic properties. It facilitated a 90 min LAD occlusion without VF. This is noteworthy, as no other animal tolerated ischemia durations longer than 40 min without VF. Clear myocardial edema and large transmural LGE were observed in pig #15, suggesting that propranolol did not drastically reduce IS, consistent with a previous study^45^.

Acetylsalicylic acid, heparin, clopidogrel and fentanyl were administered by several groups to reduce other complications like thrombosis or pain^2,52^. Pretreatment for multiple days and treatment after MI were utilized too. We refrained from this to minimize potential side effects.

A multicenter study by Kleinbongard et al. also highlighted the lack of standardization among porcine MI studies. However, the authors also emphasized that such variability overall more accurately reflects the real-world AMI patients^53^. In alignment with this perspective, the model we present in this work differs from other approaches and incorporates variability, including differences in the occlusion site. Table 2 provides an overview of the methodological features used in our study alongside those of selected previous protocols after a literature review. Representative literature was chosen based on the relevance to our research, recency and coverage of a broad range of protocol variations. The impact of those variables on study outcomes appears to depend on the specific endpoint being investigated. For example, while sex has been shown not to influence infarct size and the effect of preconditioning^44^, other studies that controlled for experimental variables reported significant differences in various outcomes^8–10^.

**Table 2:** Overview of methodological differences and outcomes between our study and key previous porcine infarction models.

	Our study	Lopez et al.^22^	Ghugre et al.^57^	Lukovic et al. ^38^	Li et al.^7^	Bönner et al.^37^	Fernández-Jiménez et al.^10^	Lubberding et al.^5^
Induction	Percutaneous intracoronary balloon							Surgical ligation/balloon occlusion
Occlusion site	Mid LAD/mid LCX	Mid LAD	Distal LAD	Mid LAD	Mid LAD	Mid LAD	Mid LAD	Mid LAD
Ischemia duration	30–90 min	90 min	45 / 90 min	90 min	90 min	90 min	20 / 40 min	60 min
Animals with AMI	20	10	8	37	44	17	20	20 open-chest, 22 closed-chest
Sex	8 males, 12 females	N.a	Female	N.a	36 males, 8 females	Female	Male	Female
Strain	German Landrace	Yucatan minipigs	Yorkshire	Domestic	Domestic	Aachen minipigs	Large White	Danish Landrace
Weight	50–70 kg	45 kg	20–25 kg	N.a	29 ± 4 kg	67 ± 9 kg	30–40 kg	51 kg
Survival rate	75%	90%	N.a	97%	95%	59%	N.a	N.a
VF rate	65%	90%	N.a	N.a	29% (all non-lethal)	76%	N.a	35% open-chest vs. 55% closed-chest
Anti-arrhythmic treatment	KCl, Mg + + , Amiodarone, (Propranolol)	Metoprolol 48 h before AMI Lidocaine, Verapamil	–	Amiodarone	Amiodarone, Lidocaine Metoprolol and Amiodarone post AMI	Amiodarone, Metoprolol	Amiodarone	–
Anti-thrombotic treatment	–	Aspirin, Clopidogrel 48 h before AMI	–	–	–	–	–	–

### EF and wall motion alterations

Reduced EF after ischemia is a consistent finding in this model^54^. In our study, the mean EF post-MI was 52%, which was lower than the baseline EF of 66% reported in a similar study using healthy German Landrace pigs, too^55^. Another study reported a mean baseline EF of 53.8%, which dropped by 10% immediately after IRI and declined further over 90 days^6^. Given this continuous EF decline over time, the relatively short interval between IRI induction and CMR in our study may explain the modest overall EF reduction observed.

We observed varying effects of ischemia on left ventricular function, as previously seen in juvenile Landrace pigs^55,56^. This variability may be attributed to differences in the occluded coronary arteries and the heart’s hyperdynamic compensatory response. Interestingly, the mean EF in animals subjected to prolonged ischemia, all measured three days post-MI, was not reduced. This challenges the validity of the measurement at this stage, as myocardial function is expected to be decreased with extending ischemia duration. Initial functional compensation by remote myocardium and early myocardial remodeling may contribute to an improved appearance of EF at this time point.

Despite these variations, an association between increasing ischemia duration and a higher frequency of wall motion abnormalities was observed in all CMR data, including in the subset analyzed three days post-MI.

### Mapping and LGE

Post-MI CMR has been performed in pigs before with various study targets^6,14,30,36^ and CMR at different time points up to 90 days post IRI-induction. CMR sequences included T1W-IR, T2-STIR, T2*w or T2w, LGE and T2 mapping^14,30,36^. Lopez et al.^22^ (Table 2) found no CMR evidence of remote myocardial fibrosis, a significant EF reduction and elevated T2 values in the infarct region up to 60 days post-AMI. Ghugre et al. (Table 2) demonstrated that quantitative CMR can differentiate between patterns of varying myocardial IRI severity in pigs. They investigated remodeling mechanisms over a six-week period using T2 and T2* mapping. Ischemia duration influenced remodeling mechanisms such as hemorrhage and MVO. T2 values remained elevated throughout the entire six-week period following a 90 min occlusion, whereas after a 45 min occlusion, they were only elevated for two weeks. The authors propose that T2 mapping may serve as a valuable tool for non-invasive monitoring of infarct healing^57^. These results suggest that the inflammatory response varies depending on the type and extent of infarction. Building on that, our study specifically aims to quantify T1 and T2 changes shortly after varying ischemia durations.

Coronary occlusion of 60–90 min induced more pronounced MI signs (edema and infarction) than occlusion of 30–45 min. The 33 min mean difference in ischemia duration between the two groups resulted in a significant difference in CMR. The presence of LGE following 60–90 min of ischemia is crucial and highlights the model’s known effectiveness in inducing a clinically relevant MI^7^. We did not detect LGE after shorter ischemic durations, whereas another study reported LGE even up to 7 d following 40 min of ischemia^10^.

Myocardial edema was also clearly dependent on the duration of occlusion. Increased T1 relaxation times were already observed after short ischemia, indicating a high sensitivity for myocardial edema shortly post-AMI. In contrast, López et al. found native T1 mapping to be unreliable for detecting infarct regions two days post-AMI, whereas T2 values were elevated in the infarct region at the same time point^22^.

The significant T2 increase in the group of 60–90 min of ischemia demonstrates the ability to detect and quantify myocardial edema after prolonged myocardial ischemia. The association of T2 changes and LGE presence in our study especially reinforces the suitability of T2 mapping to detect substantial myocardial damage. This underscores the findings of Ghugre et al., where long ischemia also resulted in greater T2 value elevations^57^. Differing from our findings, other studies observed a stronger T2 increase after ischemic durations under 60 min^30,57,58^. Fernández-Jiménez et al. (Table 2) showed increased T2 values two hours after a 20 min occlusion and reported a 28 ms increase in T2 values two hours after 40 min of ischemia^10^. This study employed different measurement techniques, assessing the individual IRI region rather than the LAD and LCX ROI. However, the discrepancy may reflect a more complex underlying tissue response and indicate limitations in T2-based differentiation between ischemic and remote myocardium in our model.

Timing is a critical factor in CMR diagnostics following MI. Myocardial edema develops early during ischemia and may fluctuate in the post-MI phase^20,58–60^. In the study by Fernández-Jiménez et al., T2 values initially increased after reperfusion but showed only a minimal elevation 24 h post-reperfusion, before rising again thereafter^10^. We avoided imaging during this transient phase of minimal edema, ensuring more reliable detection of sustained tissue alterations. Similarly, IS as assessed by LGE has been reported to decrease within the first week following MI in multiple studies^10,20^. To account for variations in the time between IRI-induction and CMR, a separate analysis was conducted on the subgroup of animals examined three days post-MI. When comparing shorter and longer ischemia durations, the findings were consistent with those of the overall analysis. This stands in accordance with a study performed in humans^21^. However, the small sample size in this subset limited statistical tests.

### Study limitations

This study included juvenile German Landrace farm pigs within a narrow age range, limiting generalizability. A further limitation is the small sample size, especially for ischemic durations over 60 min, male animals, LAD occlusions and animals undergoing CMR three days post-MI. This impaired the quality of testing for normal distribution and group comparisons.

Two different intervals between MI and CMR were used, with an imbalance in ischemia durations between them. This may have influenced CMR findings, especially regarding edema ^20,59^. The variability in occlusion site between two arteries compromises the consistency of the protocol. Information about the occlusion site was missing from one deceased animal. Pig #14 was the only animal in which coronary occlusion was resumed after a brief resuscitation, deviating from the standard protocol. In addition, propranolol was administered only to the final animal.

Regarding imaging, LGE data were missing for two pigs (#9, #11) and did not include long axis-view images. T1 map was unavailable for one pig (#7). Mapping analysis focused on a single apical short-axis slice distal to the coronary occlusion. The exact position of the slice may have differed slightly between animals. MVO was not detected but could have affected ROI measurements.

Quantification of IS and its relation to the AAR are key endpoints for evaluating the extent of IRI and the efficacy of cardioprotective interventions^53^. IS is typically quantified by LGE area in CMR^53^. In our study, LGE could have been used to investigate the time point at which substantial myocardial infarction occurs. However, conclusions regarding IS were limited by the small LGE sample size and by the use of the 17-segment model for quantification. Furthermore, the threshold for defining infarcted myocardium based on LGE was ambiguous, as mild LGE can occur in the presence of edema without actual necrosis or fibrosis^20^. In some cases, it was also challenging to distinguish subendocardial LGE from the adjacent blood pool. To assess the extent of IRI, we instead focused on quantitative CMR tissue characterization, in combination with the visual presence of LGE. By consistently measuring the same myocardial region, we ensured that larger IRI areas within the ROI also corresponded to greater elevation in mapping values. MRI findings were not correlated with ex vivo reference standards such as histochemistry for IS or AAR, which would have further validated our results.

## Conclusions

This study presents a reliable and applicable closed-chest pig model of myocardial infarction. The risk of ventricular fibrillation was particularly high in cases of prolonged ischemia, LAD occlusion, and in male animals. Our findings demonstrate that T1 and T2 mapping in combination with LGE enables the detection of IRI across a range of severities and allows differentiation between viable and non-viable myocardium. A progressive increase in edema with longer ischemic durations was revealed. The model proved especially robust and effective for ischemic durations of ≥ 60 min. Importantly, this study also provides normal reference values for T1 and T2 relaxation times in remote myocardium of juvenile German Landrace pigs. These novel insights into early post-MI CMR underscore the diagnostic value of CMR tissue characterization in MI and contribute to the growing body of knowledge in this field.

## Electronic supplementary material

Below is the link to the electronic supplementary material.

Supplementary Material 1

## Data Availability

The datasets used and/or analyzed during the current study are available from the corresponding author on reasonable request.

## Abbreviations

MRI: Magnetic resonance imaging
CMR: Cardiovascular magnetic resonance imaging
MI: Myocardial infarction
AMI: Acute myocardial infarction
CEMT: Center for Experimental Models and Transgenic Service (Freiburg, Germany)
IRI: Ischemia–reperfusion injury
AAR: Area-at-risk
IS: Infarction size
LAD: Left anterior descending coronary artery
LCX: Left circumflex coronary artery
RD: Ramus diagonalis of the LAD
VF: Ventricular fibrillation
ECG: Electrocardiogram
IV: Intravenous
CPPV: Continous positive pressure ventilation
ROI: Region of interest
Gd: Gadolinium
LGE: Late gadolinium enhancement
LV: Left ventricular
EF: Ejection fraction
AHA: American Heart Association
ESC: European Society of Cardiology
MVO: Microvascular obstruction
bw: Bodyweight
min: Minute(s)
