# Supplementary material for: Cardiac MRI of differing ischemia and reperfusion times in a myocardial infarction pig model
Source: Sci Rep. 2025 Jul 19;15:26270. doi: 10.1038/s41598-025-11390-3 (PMC12276265; doi:10.1038/s41598-025-11390-3)
Supplement: Supplementary file 1 — Supplementary Material 1 [file 41598_2025_11390_MOESM1_ESM.docx]

**Supplementary Material**

**Supplementary Table 1**

| Pig | Ischemia duration | Occluded artery | Sex | Impaired LV wall motion | EF | T1 map alteration | T2 map alteration | LGE | Comment |
| --- | --- | --- | --- | --- | --- | --- | --- | --- | --- |
| #1 | 30 min | LAD | m | positive | 55% | positive | positive | negative | resuscitated |
| #2 | 34 min | LCX | f | positive | 48% | positive | negative | negative | n/a |
| #3 | 35 min | LCX | f | positive | 42% | positive | negative | negative | n/a |
| #4 | 36 min | LCX | f | borderline | 52% | positive | positive | negative | resuscitated |
| #5 | 37 min | LCX | f | negative | 39% | positive | positive | negative | n/a |
| #6 | 37 min | LAD | m | positive | 39% | positive | positive | negative | resuscitated |
| #7 | 39 min | LAD | f | positive | 46% | n/a | negative | negative | n/a |
| #8 | 39 min | LCX | m | negative | 49% | positive | positive | negative | n/a |
| #9 | 40 min | LCX | m | negative | 64% | positive | negative | n/a | death in MRI |
| #10 | 42 min | LCX | m | borderline | 42% | negative | positive | negative | resuscitated |

**Table 1. Characteristics of pigs with MRI 2-5 h after myocardial ischemia.**

EF = left ventricular ejection fraction, LV = left ventricular, LGE = late gadolinium enhancement, LAD = left anterior descending coronary artery, LCX = left circumflex coronary artery, m = male, f = female.

**Supplementary Table 2**

| Pig | Ischemia duration | Occluded artery | Sex | Impaired LV wall motion | EF | T1 map alteration | T2 map alteration | LGE | Comment |
| --- | --- | --- | --- | --- | --- | --- | --- | --- | --- |
| #11 | 33 min | LCX | f | negative | 51% | negative | negative | n/a | n/a |
| #12 | 45 min | LCX | f | negative | 60% | positive | negative | negative | resuscitated |
| #13 | 60 min | LAD | m | negative | 74% | positive | positive | positive | resuscitated |
| #14 | 60 min | LAD | m | positive | 59% | positive | positive | positive | resuscitated |
| #15 | 90 min | LAD | f | positive | 56% | positive | positive | positive | n/a |

**Table 2. Characteristics of pigs with MRI 3 d after myocardial ischemia.**

EF = left ventricular ejection fraction, LV = left ventricular, LGE = late gadolinium enhancement, LAD = left anterior descending coronary artery, LCX = left circumflex coronary artery, m = male, f = female.

**Supplementary Table 3**

| Pig | Impaired LV wall motion site | LV T1 map alteration site | T1 map [ms] of apical LAD-ROI | T1 map [ms] of apical LCX-ROI | LV T2 map alteration site | T2 map [ms] of apical LAD-ROI | T2 map [ms] of apical LCX-ROI | LV LGE site (segments) |
| --- | --- | --- | --- | --- | --- | --- | --- | --- |
| #1 | septum and anterior (mid and apical) | septum and anterior (mid and apical) | 1335   ± 72 * | 1216   ± 101 | septum & anterior (mid and apical) | 49,7   ± 10,9 * | 39,0   ± 11,2 | n/a |
| #2 | posterior and lateral (mid and apical) | posterior and lateral (mid and apical) | 1143   ± 33 | 1239   ± 59 * | n/a | 37,3   ± 6,5 | 33,1   ± 3,9 * | n/a |
| #3 | lateral mid | posterior and lateral (apical) | 1218   ± 84 | 1265   ± 95 * | n/a | 35,9   ± 2,9 | 37,2   ± 3,4 * | n/a |
| #4 | posterior | posterior and lateral (apical) | 1225   ± 62 | 1263   ± 95 * | posterior and lateral (apical) | 41   ± 3,3 | 45,4   ± 6,9 * | n/a |
| #5 | n/a | posterior and lateral (mid and apical) | 1236   ± 63 | 1306   ± 91 * | posterior and lateral (apical) | 39,6   ± 3,9 | 42,5   ± 3,4 * | n/a |
| #6 | global, septal pronounced | septum and anterior (apical) | 1442   ± 79 * | 1362   ± 85 | septum & anterior (mid) | 42,1   ± 5,8 * | 38,1   ± 7,1 | n/a |
| #7 | n/a | n/a | n/a | n/a | n/a | 41,2   ± 9,3 * | 40,2   ± 21,5 | n/a |
| #8 | n/a | posterior and lateral (apical) | 1220   ± 97 | 1234   ± 84 * | posterior and lateral (apical) | 37,7   ± 3,8 | 41,9   ± 9,7 * | n/a |
| #9 | n/a | posterior and lateral (apical) | 1196   ± 52 | 1244   ± 128 * | n/a | 41,6   ± 6,1 | 38,9   ± 4,6 * | n/a |
| #10 | lateral | n/a | 1233   ± 74 | 1286   ± 112 * | posterior and lateral (apical) | 41,6   ± 9,4 | 45,2   ± 9,5 * | n/a |

**Table 3. Imaging characteristics of pigs with MRI 2-5 h after myocardial ischemia.**

Values are given as mean ± standard deviation, * = infarction area, LV = left ventricular, segments refer to the 17-Segment Model (AHA).

**Supplementary Table 4**

| Pig | Impaired LV wall motion site | LV T1 map alteration site | T1 map [ms] of apical LAD-ROI | T1 map [ms] of apical LCX-ROI | LV T2 map alteration site | T2 map [ms] of apical LAD-ROI | T2 map [ms] of apical LCX-ROI | LV LGE site (segments) |
| --- | --- | --- | --- | --- | --- | --- | --- | --- |
| #11 | n/a | n/a | 1275   ± 82 | 1308   ± 167 * | n/a | 39,2   ± 7,7 | 38,2   ± 5,1 * | n/a |
| #12 | n/a | posterior and lateral (apical) | 1287   ± 92 | 1397   ± 125 * | n/a | 40   ± 5,4 | 38,1   ± 5,6 * | n/a |
| #13 | n/a | septum and anterior (mid and apical) | 1411   ± 149 * | 1182   ± 117 | septum & anterior (mid and apical) | 48,5   ± 8,2 * | 38,4   ± 3,9 | septum & anterior, subendocardial (2,8,13,14) |
| #14 | septum and anterior (mid) | septum and anterior (mid and apical) | 1409   ± 71 * | 1262   ± 89 | septum & anterior (mid and apical) | 43,7   ± 5,7 * | 36,7   ± 12,5 | septum & anterior, subendocardial (8,13,14) |
| #15 | septum and anterior (mid and apical) | septum and anterior (mid and apical) | 1422   ± 133 * | 1230   ± 77 | septum & anterior (mid and apical) | 41,7   ± 5,1 * | 34   ± 3,1 | septum & anterior, transmural (8,13,14) |

**Table 4. Imaging characteristics of pigs with MRI 3 d after myocardial ischemia.**

Values are given as mean ± standard deviation, * = infarction area, LV = left ventricular, segments refer to the 17-Segment Model (AHA).
